# Multiparametric MRI-Targeted TRUS Prostate Biopsies Using Visual Registration

**DOI:** 10.1155/2014/819360

**Published:** 2014-12-01

**Authors:** Philippe Puech, Adil Ouzzane, Vianney Gaillard, Nacim Betrouni, Benoit Renard, Arnauld Villers, Laurent Lemaitre

**Affiliations:** ^1^Department of Radiology, Lille University Hospital, Lille University, 59000 Lille, France; ^2^Inserm U703, 59120 Loos, France; ^3^Department of Urology, Lille University Hospital, Lille University, 59000 Lille, France

## Abstract

Prebiopsy multiparametric prostate MRI (mp-MRI), followed by transrectal ultrasound-guided (TRUS-G) target biopsies (TB) of the prostate is a key combination for the diagnosis of clinically significant prostate cancers (CSPCa), to avoid prostate cancer (PCa) overtreatment. Several techniques are available for guiding TB to the suspicious mp-MRI targets, but the simplest, cheapest, and easiest to learn is “cognitive,” with visual registration of MRI and TRUS data. This review details the successive steps of the method (target detection, mp-MRI reporting, intermodality fusion, TRUS guidance to target, sampling simulation, sampling, TRUS session reporting, and quality insurance), how to optimize each, and the global indications of mp-MRI-targeted biopsies. We discuss the diagnostic yield of visually-registered TB in comparison with conventional biopsy, and TB performed using other registration methods.

## 1. Introduction

The positive diagnosis of prostate cancer (PCa) requires a direct sampling of the gland. This is an invasive procedure, with rare, but not negligible, potential complications [1]. However, it is a mandatory step for accurately proving, localizing, and characterizing cancer aggressiveness (Gleason score, tumour burden and extent,…) in men having a clinical of biological suspicion of PCa. Nevertheless, extended TRUS systematic posterior biopsies (SB) protocols fail identifying significant versus insignificant cancers and suffer high rate of false negatives, between 20 and 30% [2, 3]. This can easily be explained by two facts: first, by TRUS physical limitations (distance from the probe, calcifications, operator dependence), making this examination perform best in the posterior part of the peripheral zone (PZ) and poor elsewhere; despite significant technical advances [4, 5]; second, to the limitation of SB sampling to the posterior part of the PZ. The addition of target biopsies (TB) on suspicious ultrasound images [6], and the numerous attempts to optimize the biopsy protocol [7–10] have been moderately efficient but did not change the story.

Prostate MRI has long time been used to assess PCa stage only, after positive biopsies, in selected patients. The advents of new imaging techniques (dynamic contrast-enhanced (DCE) imaging; diffusion-weighted imaging (DWI)), and new imaging protocols (high-resolution external phased array coils) have radically modified the way this examination is used [11–13]. In a few years, “multiparametric” MRI (mp-MRI), including morphological, DCE, and DWI imaging has proven its accuracy and reproducibility to detect, localize, and assess the extent and aggressiveness of cancer foci within the whole gland. Several teams have highlighted its usefulness before TRUS prostate biopsies in order to detect lesions that cannot be diagnosed otherwise; either due to their location (in the “gray zone”) or to their size (small lesions underevaluated by SB) [14–22].

Various techniques have been described to guide biopsies on mp-MRI targets. The simplest is to mentally perform a “*visual registration*” of MRI and TRUS data together and, thus, aim biopsies on mp-MRI targets that would never have been noticed at TRUS otherwise. This technique is the most widely used today, because it is simple, costless, and requires no additional equipment.

In this paper, we will describe how and when* visually registered mp-MRI targeted biopsies* can be performed and the way to optimize them. We will discuss their diagnostic yield in comparison with other biopsy protocols available to date.

## 2. Technique

Comparatively to MRI “in-bore” biopsies, MRI-targeted prostate biopsies performed under TRUS guidance have three constraints: (a) they are based on mp-MRI data acquired under different circumstances (without or with an endorectal balloon), implying a different geometry and a different environment (bladder or rectal repletion); (b) they are usually performed by different physicians; (c) they are manually guided. Thus, TRUS-G mp-MRI targeted biopsies (TB) will require a good TRUS operator experience, a clear communication between physicians, and a reliable registration of mp-MRI and TRUS data.

TRUS-G mp-MRI targeted biopsies can be performed using different techniques which slightly vary, depending on the biopsy route (transrectal or transperineal), on the registration method (visual or software-assisted), and on the guidance method (manual, robot-assisted), but can be summarized into 7 common successive steps (Table 1).

### 2.1. Prebiopsy Mp-MRI Detection and Reporting of Suspicious Images

#### 2.1.1. Finding Significant Cancer

This first step is common to all MRI-targeted biopsies. Radiologists usually describe MRI targets with minimal threshold sizes (6-7 mm in the peripheral zone (PZ) and 10 mm in the transition zone (TZ)), in a standardized manner described in the 2012 ESUR guidelines, to avoid describing a myriad of suspicious images. Because of this threshold size, suspicious mp-MRI lesions (having a score ranging from 3 to 5 out of 5 on the Pi-RADS scale) are de-facto considered as suspicious of clinically significant prostate cancer (CSPCa), and several studies have emphasized the fact that prebiopsy mp-MRI targeting increases diagnosis yield of PCa and CSPCa on biopsies [22–28]. Radiologists may identify up to four different suspicious targets at prebiopsy mp-MRI (usually one or two), which may lead up to eight additional biopsy cores, depending on the number of cores sampled per target (usually 2). The number of additional biopsy cores has to be limited, because they slightly increase the morbidity and acceptability of the biopsy series and require additional histopathologic processing [29]. In our experience, a maximum of two targets per patient and of two targeted cores per target is a good compromise. When more than two lesions are described on the prebiopsy mp-MRI, we advise to guide targeted biopsies on the two lesions having the most important impact for the patient (e.g., image with a suspicion of extraprostatic or seminal spread), or the most suspicious appearance (highest Pi-RADS score) which may reflect a higher aggressivity and potentially modify patient management at the outcome of biopsies.

#### 2.1.2. Mp-MRI Reporting

As mp-MRI and TRUS-G biopsies will be performed in different rooms, at different times, and usually by two different operators having two different specialties, the quality and accuracy of mp-MRI reporting is fundamental. The decision of performing (or not) a TB on an image, the selection of the best targets, and the matching of MRI and ultrasound data will highly depend on it.

The simpler reporting is a plain-text prose description of the lesion(s), detailing its (their) location (based on the recommended 27 regions standard diagram), size (in mm) in at least 2 dimensions, MRI appearance on T2, DWI and DCE sequences, Pi-RADS suspicion score (5-point scale), and likelihood of extraprostatic spread.

**Figure 1:**
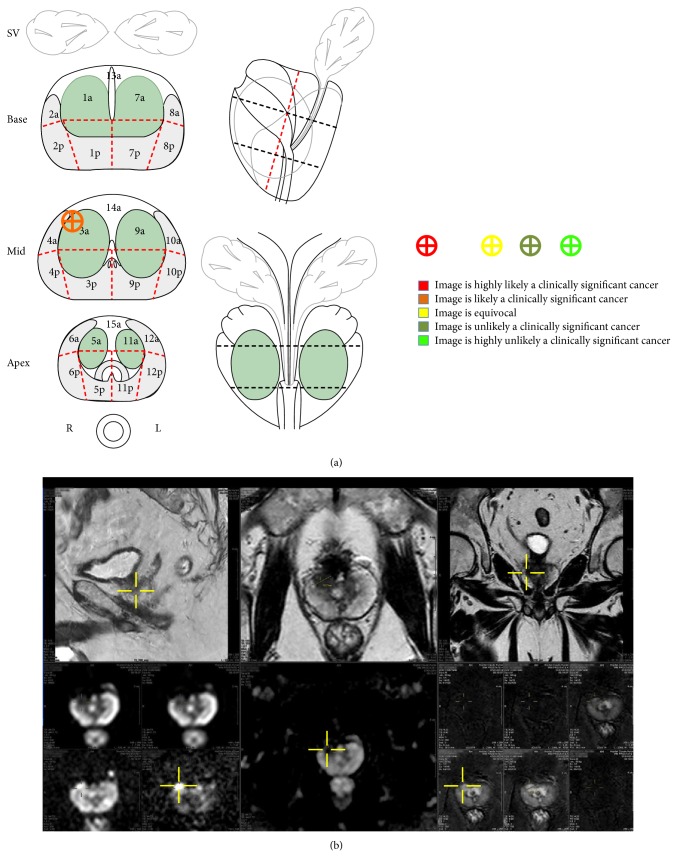
Standardized mp-MRI target reporting. 69-year-old man enrolled into active surveillance without MRI, one year ago. First AS control with prebiopsy MRI shows a suspicious (4/5) image in the anterior horn of the right PZ. Transmission of this information to the urologist performing the TB is made using the recommended standardized 27-sector diagram, common to radiologists, urologists, and pathologists in the institution. It is simply pasted at the end of the traditional text report. A screenshot of the workstation, centred on the image, is also copy/pasted in the report and saved into the PACS system. This information is available at time of TRUS biopsies. TB confirmed the diagnosis with 1 out of 2 cores positive (5 mm; Gleason 3 + 4 = 7). No SB was positive.

It is recommended to enhance the report with a graphic diagram of the prostate, on which mp-MRI targets are drawn (manually or electronically), optionally using a color scale for the Pi-RADS score (Figure 1). This schematic view of targets location, size, extent, and suspicion score allows fast selection of the most significant targets, highly appreciable during the biopsy session.

Another enhancement of the reporting is the addition of key images on the diagram for each lesion. These key images may include orthogonal projection for accurate craniocaudal localization (Figure 1).

In any event, we recommend physicians performing the TRUS-G biopsies to review the MRI examination a few minutes before the TRUS sessions on the PACS (if possible in presence of the radiologist who interpreted the case) to select targets in the best conditions.

### 2.2. Sampling of Mp-MRI Targets under TRUS Guidance

#### 2.2.1. MRI-TRUS Visual Registration

**Figure 2:**
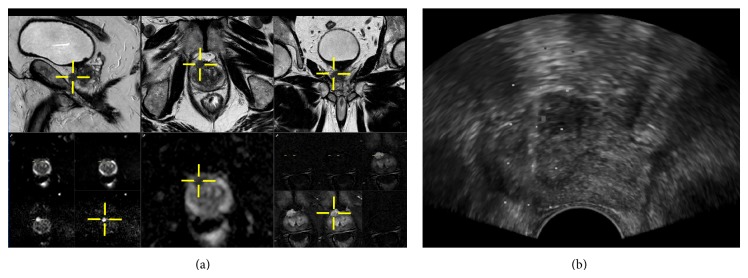
Mp-MRI targeted TRUS-G biopsy of clinically significant prostate cancer. 66-year-old patient with a PSA of 8 ng/mL. Prebiopsy mp-MRI (a) shows a 9 mm low T2 nodule (image (a); upper row), with high retriction of water diffusion and hypervascularization (image (a); lower row) in the anterior horn of the right apical PZ, ahead of the posterior 18 mm of gland sampled by SB cores. A TB was performed with knowledge of this information. It diagnosed a CSPCa (4 out of 4 positive TB; no SB was positive). Image (b) shows the trace of the needle biopsy gun inside the nodule. This lesion was aimed with visual registration, thanks to its zonal anatomy (ahead of the anterior TZ, at the edge of the anterior prostate surface), its size, and the presence of a small cyst in the right TZ (not visible on image (b), but used to locate the craniocaudal location of the lesion at time of biopsy).

**Figure 3:**
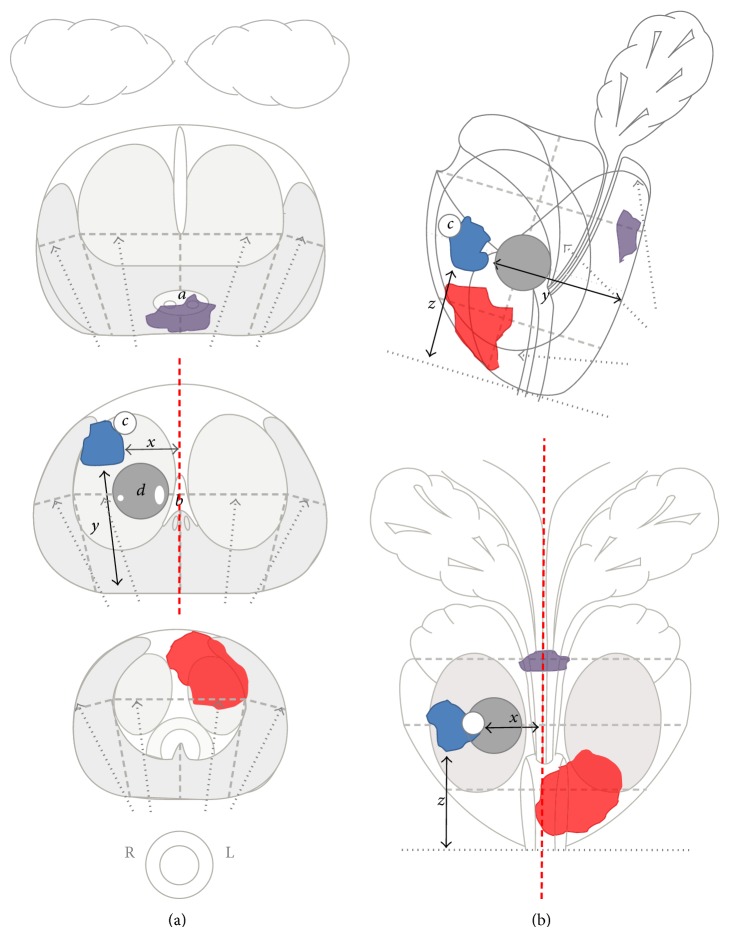
Use of anatomical and stereotactic landmarks for visual registration of 3 sample mp-MRI targets located in the “gray zone.” This figure shows three schematic representations of prostate base, midgland and apex ((a); top to bottom) sections, represented by gray dashed lines on the sagittal and coronal views ((b); top and bottom, resp.). The standard 12 systematic biopsy (SB) cores are represented by dotted arrows. The red lesion is a typical anterior apex cancer; the blue lesion is right TZ cancer, and the purple lesion a PZ cancer located on the midline. All have a clinically significant volume. Visual registration of a mp-MRI target on the TRUS image can be helped by multiple anatomical landmarks or simple three-dimensional distances, both on the craniocaudal plane (ejaculatory ducts (*a*), verumontanum (*b*), distance from the apex (*z*)), and on the axial plane (distance from the midline (*x*); distance from the posterior surface of the gland (*y*); presence of cystic (*c*) or benign nodular lesions (*d*) next to the target lesion).

Performing TB under TRUS guidance, with the visual help of the MRI images alone is called “*Visual registration,*” but is also described as “*cognitive registration*” or “*cognitive fusion*” in the literature: the TRUS operator mentally relocates the target detected on the prebiopsy mp-MRI, based on its zonal topography and on anatomical landmarks that may exist beside the lesion (cyst, BPH nodule, calcification,…) (Figures 2 and 3). This is quick and easy for physicians trained at both MRI and TRUS imaging, but may be tricky for others, which is the reason why several ultrasound manufacturers have developed specific software registration techniques to help fusion between modalities (*software registration*). Measuring the target's location from the prostate apex, lateral and posterior sides, or urethra can also help spatial registration of the target (Figure 3). Visual registration is easier if MRI data is available in a separate workstation beside the ultrasound device, allowing the operator to review the MRI anatomy in T2-w sequences, relocate the target more precisely, check anatomical landmarks, and perform distance measurements described above. If the physician performing the biopsies did not interpret the MRI, he will also take benefit of a schematic interpretation report (as described in step 2). Usually, visual registration of a mp-MRI target leads to the detection of a corresponding TRUS target. Usually, this “registered” TRUS target is a hypoechoic nodule or area, of mild or high contrast with the surrounding tissue. Less frequently, it can simply not be distinguished from the rest of the gland.

#### 2.2.2. Guidance to the Target(s), Simulation, and Core Sampling

When the physician has located the visually registered TRUS target, he has to guide the biopsy needle gun to the target. This guidance is usually helped by the overlay of a dotted line symbolizing the needle direction on the real-time image.

Targets located in the PZ will be easy to aim at, because they are immediately located at the tip of the biopsy needle guide. Inversely, those located in the TZ will require a small learning curve: in a couple of seconds, the operator has to detect the TRUS target (step 3) with freehand motion, manually lock the probe position on the target, insert the needle into the gland, advance beyond the peripheral zone, stop the needle at the contact of the target, check that the needle will not transfix the gland, then trigger the semiautomatic needle gun. Depending on the target's depth and on the gland elasticity, the prostate or the probe may slightly move during this process, and this explains why TB may not sample the target as accurately as desired and have to be repeated at least twice in order to maximize the chance of sampling a target correctly.

**Figure 4:**
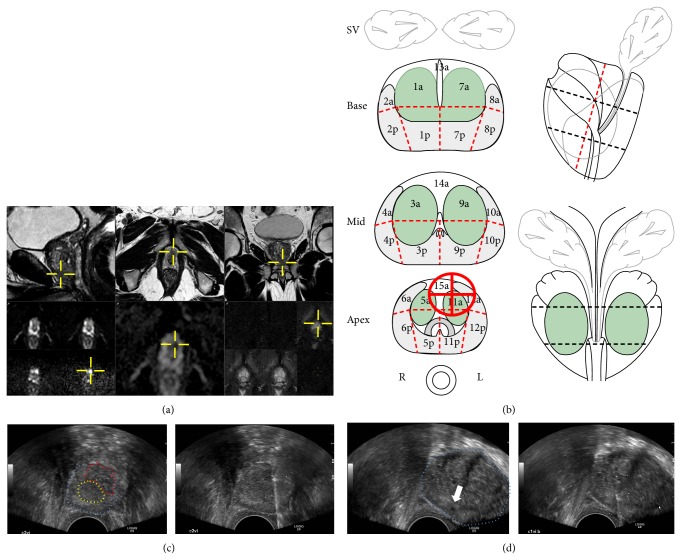
TRUS-G biopsy of two lesions requiring special needle orientation and experience for aiming TB. This 62-year-old man with a PSA of 8.25 ng/mL referred to our centre because persisting elevated PSA after 2 biopsy sessions. Last biopsy showed 2 positive cores at the right apex (2 mm each, 3 + 3 = 6). Prebiopsy MRI (a) shows a 13 mm highly suspicious (5/5) image at the extreme apex (AFMS). MRI report (b), as well as the zonal location of this lesion (extreme apex, large urethral contact, size) helped its detection at TRUS (c). This lesion (red-dotted; image (b)-left) could not be detected at TRUS imaging. Core sampling (labeled C2-VI) was performed by brushing the urethral sphincter (yellow-dotted) to avoid its perforation. A hypoechoic image ((d); left image white arrowhead) was visible at TRUS, but not described at prebiopsy MRI. It was sampled (label C1-VI) with high probe angulation (prostate contour is blue-dotted), and simulation of the needle trajectory, in order to avoid urethral or ejaculatory duct damage ((d); right image showing the needle trace). Each lesion was sampled by 2 TB. AFMS targets were both positive with 14 and 13 mm of adenocarcinoma Gleason 3 + 4 = 7; midline lesion (C2vi) TB were both negative.

A rapid simulation of the biopsy path should be done, to avoid transfixion through the bladder or urethra. The operator has to check that there is no risk of bladder wall or urethra wound in the 20 mm beyond the tip of the needle. This may happen for TB located at base or for lesion located at the anterior apex (e.g., in the anterior fibromuscular stroma (AFMS)). In this case, freehand guidance of the needle to the target can be performed by brushing the urethral sphincter (thick hypeoechoic rim), then angulating the probe medially to aim the lesion. Some targets located on the midline may also be difficult to aim due to the lateralization of the needle guide on the ultrasound probe. Applying a high angulation on the probe and performing a TB almost in parallel to the posterior prostate surface can solve this issue (Figure 4).

Guidance is the key step to the quality of TRUS-G TB (with visual or software registration). It can be improved by dedicating a physician to the probe manipulation and another one to cores sampling. An alternative option is to use a mechanical arm to lock the probe on the TRUS target, in order to handle the biopsy gun comfortably [30, 31] and perform multiple cores exactly in the same location.

### 2.3. TRUS Biopsy Report

A standardized report of the biopsy session should be provided, including detailed notification of SBs and TBs that were performed. It can be done in the same manner as for MRI, using a standardized 27-sectors diagram of the gland, including drawings (freehand or computerized) of the sampled lesions, which may be different than those described at mp-MRI. It is also important to report how good the TRUS TB matched the mp-MRI one (e.g., visibility of the lesion). All this information will be useful for analysing final histopathology results. In case of positive TB, physicians will be able to compare core cancer length with the TRUS ballistic and determine the likelihood of having sampled a significant cancer. In case of negative sampling, biopsy ballistic report will help decide whether the lesion was a false mp-MRI positive or a significant image that may have not been correctly sampled.

## 3. Indications of Mp-MRI Targeted TRUS Biopsies

Prebiopsy MRI was first proposed in patients after a negative first round of biopsies. This attitude was quickly suggested [32, 33], because of a high rate of second-round TB positivity, similar to that patients under active surveillance reclassified by mp-MRI (up to 37%) [34–37]. Indeed, cancers underdiagnosed by SB, and well detected by mp-MRI guided TB are located in a “gray zone” composed of the anterior part of the transition zone (TZ), the AFMS, the lateral and anterior horns and extreme parts of the peripheral zone (PZ) [12, 38, 39]. This attitude may quickly be recommended [19, 40] especially since a study by de Rooij et al. showed that the cost-effectiveness of a biopsy strategy including MR imaging was comparable to the TRUS biopsy strategy [41], but with a better overall quality of life and significantly less invasive procedures.

New treatment options (including focal therapy) and active surveillance (AS) highly depend on imaging results. In a series of 388 consecutive men eligible for AS, Vargas et al. have shown that a negative MRI (Pi-RADS scores 1-2) had an excellent (0.96–1) negative predictive value of CSPCa at confirmation biopsies, whereas a positive MRI (scores 3–5) was highly sensitive for upgrading on confirmation biopsies [42]. Sonn et al. recently proved that TB was 3 times more likely to identify cancer than SB (21% versus 7%), and found CSPCa in 38% of men initially enrolled for AS or with prior negative biopsies [43]. Same figure for Marliere et al. who found 39% of patients reclassified thanks to mp-MRI guided TRUS biopsies alone in a series of 41 patients [44].

Consequently, main clinical indications of prebiopsy mp-MRI combined with TRUS-G TBs (visually or electronically registered) are (a) diagnosis of CSPCa in patients with a clinical or biological suspicion of PCa (first or *n*th round); (b) diagnosis of recurrences after prostatectomy. In the future, TB may also be used to follow up selected patients having a low risk cancer and a potentially low benefit of radical treatment or willing to postpone invasive procedures due to functional risks.

## 4. Discussion

The evolution of prostate MR imaging has enabled its use before biopsies to detect cancers located in areas usually undersampled (anterior TZ, AFMS, anterior horns of the PZ, extreme apex, and base) by SB protocols and poorly detected at TRUS imaging. Consequently, in many centres, the practice of prostate biopsy has slightly evolved to prebiopsy mp-MRI, followed by SB and optional TB in case of suspicious mp-MRI target.

There are three broad categories of mp-MRI TB guidance under TRUS imaging: (a) cognitive fusion using “visual” registration (TB-VI); (b) software-assisted fusion using rigid registration (TB-FUr) (not changing MRI or TRUS data geometry) [45–47]; (c) software-assisted fusion using elastic registration (TB-FUe) (usually requiring prostate contour drawing or segmentation in both modalities, and a software fusion of the two virtual gland objects) [48]. Aside of these 3 techniques, TB guidance within the MR scanner (also called “in-bore targeting”), first described in 2000 [49], remains marginal to-date, because of cost, efficiency, and availability in comparison with TRUS imaging.

### 4.1. Assets

Cognitive fusion with visual registration (TB-VI) is the oldest, simplest, fastest, and cheapest technique. It was described in the mid 2000 [16, 50–53], in centres practicing MRI before biopsies, having also a good experience in TRUS imaging. It is now widely used worldwide [54], because it requires no additional software, adds no extra-time to the biopsy procedure, and can be performed by any physician already trained at TRUS biopsies, in an office-based procedure. Visual registration requires a good knowledge of prostate zonal anatomy, and either the ability to review MRI data just before the biopsy session, or a detailed transmission of mp-MRI targets location, or best, of both. Visual registration is also applicable for the guidance of TB on suspicious images of recurrence after prostatectomy, which is not the case for techniques requiring the fusion of two prostate volumes.

### 4.2. What Is the Added-Value of TB with Visual Registration?

In an extensive review written in 2011, Moore et al. [54] found out that a mean of 66% of men with a mp-MRI target have positive TB and that combining TB with SB leads to detection of 43% of CSPCa. They identified 18 studies in the literature, having added TB to SB between 1999 and 2011. None assessed the diagnostic yield of PCa from TBs alone, and few compare the detection of CSPCa in SB versus TB cores in the same cohort of men, as recommended by the Standards of Reporting for MRI-Targeted biopsy studies (START) consensus panel [55]. Our team [25] investigated both situations, showing, in a prospective series of 95 patients who had a suspicious image at mp-MRI, that individual SB and TB positivity rates for PCa were 59% and 69%, respectively (*P* = 0.033), and that sampling quality was better (maximum cancer length per core; Gleason grade) for TBs, regardless of using visual (TB-VI) or software assisted registration (TB-FUr). Unpublished results from this study, as well as a work of Labanaris et al., show that combination of SB and TB-VI yield 73–75% [53]. This has to be compared with the traditional 50% of positivity of SB published in the literature. Aside of the single study in the literature that found no statistically significant difference between TB-VI and SB [22], our work [25] and several others since [23, 24, 56], have shown that TB-VI increase diagnostic yield of CSPCa. This suggests that TBs alone, performed with the help of prebiopsy mp-MRI, may perform better than “blind” SBs alone to detect significant cancer and to assess cancer aggressiveness. Nevertheless, no study to date has proved that TBs alone perform better than the combination of SB and TB.

### 4.3. How Good Is Visual Registration in Comparison with Software-Assisted Registration?

It is not possible to compare geometric accuracies of cognitive and software fusion techniques together, but some studies have compared the diagnostic yield of PCa from TBs performed using both visual (TB-VI) and software-assisted (TB-FU) fusion techniques, in terms of positivity for cancer, cancer length per core, or Gleason score: Mouraviev et al. published a series of 32 patients and found significantly different detection rates of 33.3% versus 46.2%, and sensitivities of 45.5% versus 61.9% for TB-VI and TB-FUr, respectively [57]. Later, Wysock et al. published another study comparing TB-VI and TB-FUr techniques [24]. In this monocentric prospective series of 172 targets in 125 men, they found that TB-FUr performed better than TB-VI for detecting high grade cancer (Gleason ≥ 7; *P* = 0.0523), and an increase in diagnostic rate for TB-FUr, significant by target analysis (32% versus 26.7%, resp.; *P* = 0.1374), but not by patient analysis (36% versus 32%, resp.; *P* = 0.3588). Same figure for Delongchamps et al. who did 3 groups and found statistically significant differences between TB-VI and TB-FUr, and between TB-VI and TB-FUe [22]. Recently, [25] our team prospectively compared TBs performed manually using visual registration (TB-VI), TBs performed manually using software-assisted fusion by rigid registration (TB-FUr), with 2 cores per technique, and SB performed by another physician without knowledge of MRI data. In this group of 79 patients having a suspicious image at prebiopsy mp-MRI, 47% and 53% of TBs were positive for cancer, respectively, with no difference in subgroups of posterior, anterior, or smallest (<10 mm) mp-MRI targets, contrarily to our initial impression that TB-FUr could be better for smaller or very anterior lesions, hardly visible at TRUS imaging (Figure 4). Mean longest cancer core lengths were 7.27 mm and 7.30 mm for TB-VI and TB-FUr, again with no statistically significant difference. For Gleason analysis, TB-VI and TB-FUr performed equally in 79% of cases, and TB-FUr showed higher GS in 4 patients, but lower on 3 targets. Overall, we did not prove a statistically significant difference between visual and software-assisted registration techniques. Limitations discussed in the literature are that numerous variables influence the diagnostic yield of TBs, and may modify the outcome of the biopsies: the scoring technique of mp-MRI targets; their size, the number of cores per TB, and even the mean core length. We believe that the quality of communication between the physician who interpreted the MRI and the one performing the TRUS-G biopsies (standardized report, interdisciplinary meeting, discussion of the case, presence of the radiologist during the biopsy session,…), as well as their mutual experience are of major importance.

### 4.4. Limitations

**Figure 5:**
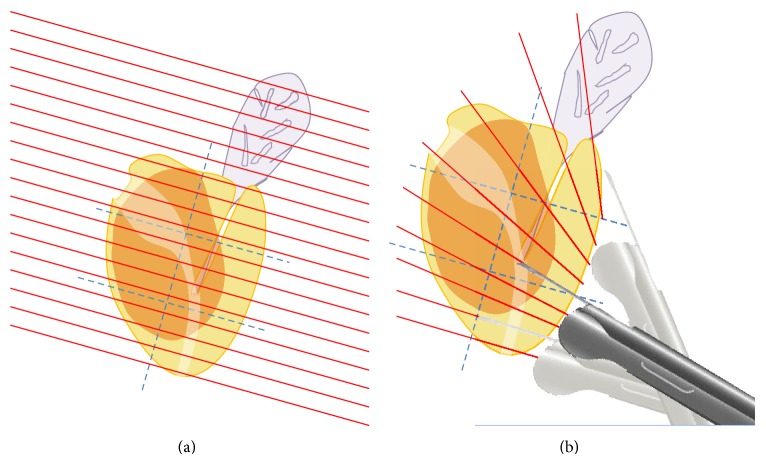
Comparison of MR and TRUS imaging acquisition planes of the prostate. MRI (a) and TRUS (b) imaging are performed with different acquisition planes. MR imaging is usually acquired perpendicularly to the prostate wall (or strictly transverse), with a slice thickness varying from 2.5 to 5 mm, homogeneously distributed (red lines overlaid on image (a)). TRUS imaging is acquired using an endorectal probe inserted in rectum, through the anal canal which is a fixed point that limitates probe translation and angulation. Hence, TRUS images acquired at the apex will often match MRI, whereas those acquired at the base will require a registration step (visual or computer-assisted) to match MRI.

Some disadvantages of TB-VI should be highlighted: (a) they are operator dependent. TB-VI require more experience and precision than SB, with good knowledge of both MRI and ultrasound semiology to be able to match MRI and TRUS images; (b) diagnostic accuracy might vary depending on lesion visibility on TRUS imaging, and lesion location, as TRUS and MRI do not have the same exploration planes: lesions located at the inferior part of the gland, MRI and TRUS axial sections will be visible on slices acquired on nearly identical planes, whereas images located at the upper part of the gland, or anteriorly, will not (Figure 5). Both limitations might be reduced by training and simulation. New promising simulation tools have recently been described for that purpose [58, 59]. (c) Last, there is no per- or postprocedure quality control. In case of negative TB, the interpretation of this result is hazardous, because there is no way to be sure that the target was hit and that a second TB session (in case of high clinical or MRI suspicion) can sample the same image a second time. A good approach is to save a screenshot of the needle trace for each core of TBs (Figures 2 and 4). In the future, it may be possible to track the position of the needle on the image and locate TB cores in a reconstructed 3D TRUS series of images.

## 5. Conclusion

Prebiopsy mpMRI combined with TRUS-guided target biopsies of the prostate is a major step forward over systematic biopsies alone. It is becoming a key for the diagnosis of prostate cancer, because it detects significant cancers in areas usually undersampled by systematic biopsies. Those biopsies can be performed using visual or software-assisted registration of MRI and US data. In comparison with other MRI-US fusion techniques, visual registration is easier to learn, cheapest and simpler, making it compatible with daily office practice and a potential inclusion in the standard diagnostic pathway of PCa.

## Figures and Tables

**Table 1:** Seven-step protocol for prostate biopsies performed under TRUS guidance with mp-MRI targeting.

	Actors	Steps	Summary	Description
MRI room	Radiologist	1	Target detection	Detection of mp-MRI lesions having a cancer suspicion score ≥3 and a significant size. A mean of two targets is a good compromise, with a primary target clearly identified.
		2	Target reporting	Transmission of intelligible and accurate information to the physician that will perform the biopsy procedure.

TRUS biopsy room	Urologist/radiologist/both	3	Intermodality fusion	Registration (or “fusion”) of static, asynchronous, and multiparametric MRI data with that of a real-time and dynamic TRUS data.
		4	TRUS guidance to target(s)	Guidance of the biopsy needle gun to the correct location of the mp-MRI target within the TRUS image volume.
		5	Sampling simulation	Simulation the sampling helps the physician assess the quality of the sampled core, as well as the safety of the sampling.
		6	Tissue sampling	Tissue sampling is usually performed using a semiautomatic needle biopsy gun triggered by the physician.
		7	TRUS biopsy report and quality insurance	Reporting of the biopsy procedure, including the location of this additional cores and the correspondence with suspicious mp-MRI image.

## Abbreviations

AFMS: Anterior fibromuscular stroma of the prostate
BPH: Benign prostatic hyperplasia
CSPCa: Clinically significant prostate cancer
DCE: Dynamic contrast-enhanced MR imaging
DWI: Diffusion-weighted imaging
ESUR: European Society of Uroradiology
mp-MRI: Multiparametric prostate MRI
PACS: Picture archiving and communication system
PCa: Prostate cancer
PZ: Peripheral Zone of the prostate
SB: Extended TRUS systematic posterior biopsies
TB: Target biopsy
TB-FU: TBs performed manually under TRUS-G with software-assisted registration (rigid or elastic)
TB-FUe: TBs performed manually under TRUS-G with software-assisted registration (elastic)
TB-FUr: TBs performed manually under TRUS-G with software-assisted registration (rigid/isometric)
TB-VI: TBs performed manually under TRUS-G with visual registration
TRUS-G: Transrectal ultrasound-guided
TZ: Transition Zone of the prostate.
